# Long-term survival in a patient with repeated resections for lung metastasis after hepatectomy for ruptured hepatocellular carcinoma: a case report

**DOI:** 10.1186/1752-1947-2-222

**Published:** 2008-06-30

**Authors:** Kai-Lun Shih, Yang-Yuan Chen, Tsung-Han Teng, Maw-Soan Soon

**Affiliations:** 1 Department of Gastroenterology Changhua Christian Hospital, Changhua, Taiwan; 2 Department of Pathology, Changhua Christian Hospital, Changhua, Taiwan

## Abstract

**Introduction:**

Tumor rupture and pulmonary metastasis in patients with hepatocellular carcinoma are both associated with poor prognosis and treatment strategies are controversial.

**Case presentation:**

Here we report a 50-year-old man with survival of over 90 months after undergoing an extended right lobectomy for a ruptured hepatocellular carcinoma and then repeated resections for pulmonary metastasis during the followup period.

**Conclusion:**

This case report shows that surgical resection can be an effective treatment for patients with both ruptured hepatocellular carcinoma and pulmonary recurrences.

## Introduction

Hepatocellular carcinoma (HCC) is the most common primary hepatic tumor and one of the most common cancers worldwide. Spontaneous rupture is a life-threatening complication of HCC. The overall incidence of spontaneous rupture of HCC varies from 5% to 26%, with a mortality rate of up to 67%, especially in patients with poor liver function [1-4].

The treatment of a ruptured HCC is controversial. Previous studies have suggested that emergency liver resection is feasible in patients with a small tumor and satisfactory liver function (Child-Pugh A or B grade). Surgical resection is currently the only way to achieve long-term survival [5].

The lung is the most common site of metastasis in patients with HCC. These are often unresectable as most pulmonary metastases are multiple [6]. Nevertheless, some studies have revealed that surgical resection of pulmonary metastases from HCC may prolong survival in selected patients [7-9]. Ruptured HCC often exacerbates the risk of disseminated intraperitoneal metastases, and previous studies have suggested long-term survival may be possible with aggressive surgical treatment, even if intraperitoneal metastases develop [10,11]. However, to our knowledge there has been no report of a patient who has undergone resections of pulmonary metastasis after hepatectomy for a ruptured HCC. Here we report a rare case of long-term survival after three pulmonary metastasectomies following hepatectomy for a ruptured HCC.

## Case presentation

**Figure 1:**
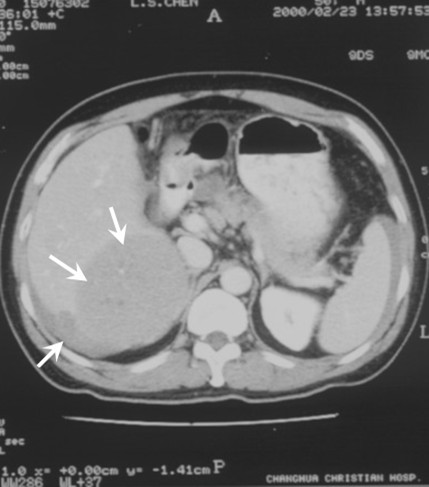
Abdominal computed tomography on admission showing a mass in Couinaud segment 7 and segment 8 of the liver and intraperitoneal fluid.

On 23 February 2000, a 50-year-old man was referred to our hospital because of progressive abdominal pain for 3 hours. The patient's initial blood pressure was 88/58 mmHg and his heart rate was 109 beats/minute. Laboratory data were within normal limits except for the hemoglobin and alanine aminotransferase, which were 8.8 g/dl, and 145 U/l, respectively. After transfusion of 2 units of packed red blood cells and 4 units of fresh frozen plasma, the patient's blood pressure was 112/63 mmHg and his heart rate was 75 beats/minute. Abdominal computed tomography (CT) on admission showed a 12 × 12.5 × 5 cm^3 ^mass in Couinaud segments 7 and 8 and ascites accumulation (Figure 1). Angiography revealed dilated, tortuous and displaced arterial tumor feeders with neovasculatures showing a disorganized pattern over the right lobe of the liver. This was consistent with HCC. Other laboratory data revealed positive hepatitis B virus surface antigen and antibodies against hepatitis C virus. Serum alpha-fetoprotein level was less than 20 ng/ml.

The patient underwent a right extended lobectomy, with partial resection of the diaphragm (about 4 × 4 cm^2^) and cholecystectomy, on 26 February 2000. The tumor was resected with margins greater than or equal to 1 cm. Six hundred cubic centimetres of bloody ascites were removed during surgery.

**Figure 2:**
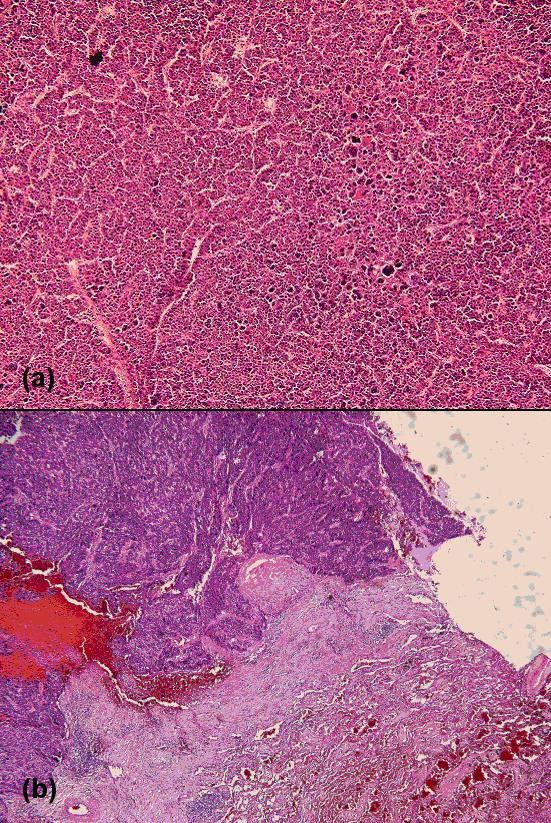
2a: Polygonal cells with higher N/C (nucleus-to-cytoplasm) ratio than normal, abundant granular eosinophilic cytoplasm, round nuclei with coarse chromatin and an area of giant cell (original magnification ×100). -2b: Metastatic hepatocellular carcinoma in lung parenchyma (original magnification ×40).

Histopathological examination revealed a poorly differentiated HCC (Figure 2a). Direct invasion to the resected diaphragm was seen, although the microscopic surgical margins were unremarkable.

The patient's postoperative course was smooth and he was discharged on 13 March 2000. After surgery, he had regular followups with serum alpha-fetoprotein levels, chest radiographs, and abdominal ultrasonography every three months in our hospital. A small intrahepatic recurrence was found and treated twice by ultrasound-guided percutaneous alcohol injection.

The patient presented with mild hemoptysis 30 months after the hepatectomy, and follow-up chest CT demonstrated a metastasis in the right lower lobe the of lungs. The metastasis was removed with wedge resection measuring 2 × 1 × 1 cm^3^. Histopathological examination confirmed the presence of metastatic HCC (Figure 2b).

A follow-up chest radiograph and CT revealed a 2.9 cm solitary metastasis in the right upper lobe of the lung without any sign of liver recurrence 52 months after hepatectomy. A wedge resection of the right upper lobe of the lung was performed through a thoracotomy and the tumor was confirmed as a metastasis of HCC.

**Figure 3:**
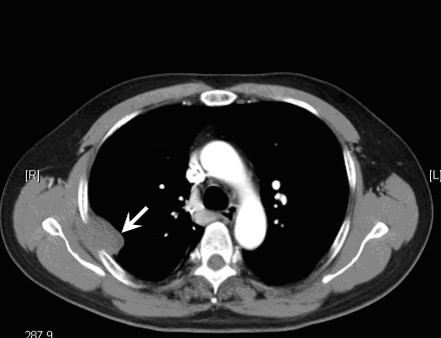
Chest computed tomography showing lung metastasis with pleural seeding 80 months after hepatectomy.

A further follow-up chest CT scan disclosed tumor recurrence in the right upper lobe of the lung and pleural seeding 80 months after hepatectomy (Figure 3). The serum alpha-fetoprotein level was 39.14 ng/ml. The patient underwent surgery again in December 2006 and these tumors were removed with combined resection of the right upper pulmonary lobe and right chest wall with the fourth and fifth ribs. Histopathological examination confirmed the presence of metastatic HCC. After surgery, the serum alpha-fetoprotein level decreased to 1.96 ng/ml in February 2007.

No intrahepatic recurrence of HCC was found after the last percutaneous alcohol injection of the liver and the patient has remained disease-free for 10 months since the last resection of pulmonary metastases, i.e. 90 months after the initial hepatectomy for a ruptured HCC.

## Discussion

The exact mechanism of spontaneous HCC rupture is still unknown although hypotheses include rapid growth of tumor with necrosis, erosion of a vessel, occlusion of the hepatic veins by a tumor thrombus, and coagulopathy [12]. The prognosis of ruptured HCC is poor because many patients have advanced disease at the time of rupture and may also have cirrhosis. Malignant cells sometimes disseminate into the peritoneal cavity contributing to poor prognosis [1]. There have been few reports of successful resection of peritoneal and pleural disseminated metastases caused by a ruptured HCC [11,13].

In our patient, HCC with direct invasion to the diaphragm was seen and was resected with clear margins, and intrahepatic recurrence was controlled by local ablation. Pulmonary metastases were diagnosed 30, 52, and 80 months after hepatectomy and were removed by repeated wedge resections.

The lung is the most common site for extrahepatic spread of HCC and leads to a poor prognosis [14]. Some authors suggest that an extrahepatic metastasis should be treated medically [15]. Previous literature described four criteria for pulmonary metastasectomy: [1] the patient must be a good risk for surgical intervention; [2] the primary malignancy must be controlled; [3] there should be no other extrapulmonary metastasis, or, if present, it can be controlled by surgery or another treatment modality; and [4] the pulmonary metastases are believed to be completely resectable [16,17].

Some reports support the effectiveness of pulmonary resection of metastases from HCC. Tomimaru et al. [9] reported that surgical resection for pulmonary metastasis from HCC is beneficial on the condition that the number of lung metastases is limited to one or two, and any intrahepatic recurrence is well managed.

Factors for good prognosis after pulmonary metastasectomy of HCC include a patient's disease-free interval greater than 12 months and alpha-fetoprotein levels less than 500 ng/ml [8]. For our patient, the number of lung metastases during the third metastasectomy was two, and his alpha-fetoprotein levels returned to normal levels soon after the operation.

Few reports described long-term survival after resection of pulmonary metastases from HCC [18]. Our case report suggests that for a patient with HCC and pulmonary metastases, surgical resection for lung metastases can be effective if the number of lung metastases is less than two.

## Conclusion

This case report suggests that surgical resection can be an effective treatment for patients with both ruptured HCC and pulmonary recurrences. When intrahepatic recurrences are not present, a previous episode of HCC rupture is not a contraindication for pulmonary metastasectomy in patients with HCC.

## Abbreviations

CT: computed tomography; HCC: hepatocellular carcinoma.

## Competing interests

The authors declare that they have no competing interests.

## Authors' contributions

KLS and YYC examined the patient, reviewed the literature, and wrote the manuscript. The manuscript was reviewed and edited by YYC and MSS. All authors read and approved the final manuscript.

## Consent

Written informed consent was obtained from the patient for publication of this case report and any accompanying images. A copy of the written consent is available for review by the Editor-in-Chief of this journal.
